# Early Detection of Treatment Response to Mavacamten in Hypertrophic Obstructive Cardiomyopathy With Severe Mitral Regurgitation Using Magnetocardiography

**DOI:** 10.1002/ccr3.70667

**Published:** 2025-08-20

**Authors:** Phillip Suwalski, Gamze Satilmis, Markus Reinthaler, Ulf Landmesser, Bettina Heidecker

**Affiliations:** ^1^ Deutsches Herzzentrum der Charité – Universitätsmedizin Berlin Corporate Member of Freie Universität Berlin and Humboldt – Universität zu Berlin Berlin Germany; ^2^ Institute of Biomaterial Science and Berlin‐Brandenburg Centre for Regenerative Therapies (BCRT) Hereon Helmholtz‐Zentrum Geesthacht Teltow Germany; ^3^ Berlin Institute of Health (BIH) at Charité Berlin Germany; ^4^ DZHK (German Centre for Cardiovascular Research) Partner Site Berlin Berlin Germany

**Keywords:** cardiology, cardiovascular disorders, chronic diseases, complementary and alternative medicine, radiology and imaging

## Abstract

Recent evidence highlights the efficacy of mavacamten in reducing left ventricular outflow tract (LVOT) gradients in patients with hypertrophic cardiomyopathy (HCM), which is illustrated in this case. Additionally, the potential of magnetocardiography (MCG) as a novel, objective diagnostic and monitoring tool for nonischemic cardiomyopathies is demonstrated.

## 1 Introduction

Hypertrophic cardiomyopathy (HCM), with an estimated prevalence of 0.2%, is overall rare [1], yet it is one of the more common nonischemic cardiomyopathies, often caused by genetic variants [2]. About 60% of HCM cases are inherited in an autosomal‐dominant manner, primarily involving sarcomere‐coding genes [1], which leads to myocardial hypercontractility progressing to stiffness, reduced compliance, fibrosis, and left ventricular hypertrophy [2]. Depending on the severity, myocardial hypertrophy can lead to an obstruction of the left ventricular outflow tract (LVOT) in two‐thirds of the patients [1]. According to recent studies, the disease occurs worldwide with a similar prevalence. However, there are significant biological sex differences [3]. Compared to men, women are typically older and present with more severe symptoms at the initial diagnosis, potentially also having a lower survival rate [2].

Phenotypically, affected individuals are often asymptomatic in early stages, but may develop dyspnea, heart failure, conduction disturbances, arrhythmias, or experience sudden cardiac death (SCD [1]). In patients younger than 35 years and well‐trained athletes, HCM is considered the most common cause of SCDs [1, 2].

The diagnosis of HCM includes echocardiography and cardiovascular magnetic resonance (CMR) for detection of fibrosis. The focus is on wall morphology, with a thickness exceeding 15 mm within the left ventricle being indicative of HCM, if no other etiology is identified [3]. Using late gadolinium enhancement (LGE) in CMR, inflammatory or fibrotic myocardial tissue can be visualized to assess risk for adverse cardiovascular events [3].

On electrocardiogram (ECG), some patients may exhibit rhythm disturbances such as atrial fibrillation or non‐sustained ventricular tachycardia (NSVT), which may indicate a higher risk of ventricular fibrillation and SCD [1, 3].

Endomyocardial biopsy (EMB) and genetic testing for disease‐related variants, particularly sarcomeric genes [1, 2], help identify the specific etiology, with approximately half of the cases demonstrating an autosomal‐dominant inheritance pattern [4]. Furthermore, viral, bacterial, and other genetic causes leading to cardiomyopathy, such as cardiac amyloidosis, Danon disease, or Fabry disease, should be excluded. Despite the importance of genetic testing, positive results cannot provide exact predictions about patients' prognosis or outcome [3]. Furthermore, about half of the patients with suspicion of HCM do not show any disease‐causing variant, which is why these cases must be assessed with other diagnostic criteria, as outlined above [4].

In the presented case, magnetocardiography (MCG) was used as an additional diagnostic screening tool for cardiomyopathies, by evaluating the electromagnetic field and to monitor the clinical course and treatment response [5].

Until recently, beta‐blockers and calcium inhibitors were the primary therapeutic options for treating HCM. In 2022, the U.S. “Food and Drug Administration” (FDA), and in 2023, the “European Medicines Agency” (EMA) approved the cardiac myosin inhibitor mavacamten, which binds allosterically and selectively reversibly to myosin‐ATPase [3]. This reduces the formation of myosin‐actin crossbridges in myocardial cells, leading to a reduction in contractility. Thus, it directly addresses the pathophysiology of HCM, reduces the obstruction in the left ventricle, and improves cardiac filling [4]. Mavacamten is currently approved for patients with HCM, New York Heart Association (NYHA) Class II–III, left ventricular ejection fraction (LVEF) ≥ 55%, and an LVOT peak gradient ≥ 50 mmHg at rest or with provocation [2]. As reported by Keam et al., patients in a Phase 3, randomized, placebo‐controlled study were found to have a decrease in LVOT obstruction and left ventricular mass from Week 4 of therapy with mavacamten onwards. About 27% of patients receiving mavacamten had a decreased LVOT gradient of < 30 mmHg and reached NYHA Class I [2]. About 80.9% of patients had a complete resolution of systolic anterior motion (SAM [2]). Furthermore, it has been shown that mavacamten successfully reduced the LVOT gradient in patients needing septum reduction therapy (SRT) otherwise to < 50 mmHg in about 74% [2, 6]. At our institution, we follow the current mavacamten protocol, published by the EMA [7]. Prior to initiating mavacamten, the CYP2C19 metabolizer phenotype must be determined, distinguishing between slow and rapid metabolizers. Slow metabolizers should start with an initial dose of 2.5 mg/day, while rapid metabolizers can begin at 5 mg/day. Regardless of the metabolizer phenotype, all patients should undergo regular assessments to monitor the LVOT gradient, LVEF, treatment response, and potential side effects [7].

Commonly used invasive methods to eliminate hypertrophic myocardium are transcoronary ablation of septal hypertrophy (TASH) or ventricular septal myectomy [4]. In addition to the inherent risks of any invasive cardiac procedure, both procedures can cause life‐threatening arrhythmias, whereby this risk is higher for myectomy than for TASH [8].

This case report presents the case of a 29‐year‐old patient who has suffered from HCM since the age of 15 and has been successfully treated with mavacamten since early 2024.

The effectiveness of mavacamten in HCM management is highlighted. Additionally, it demonstrates the potential of MCG as a rapid, objective tool to screen cardiomyopathies, including HCM, and monitor treatment response [5].

## 2 Case Presentation

We report the case of a 29‐year‐old male patient with a history of HCM, first diagnosed at the age of 15, who presented to our hospital for further diagnostic evaluation. The patient's previous medical history included von Willebrand disease, hypothyroidism, and migraine. With regard to family history, two of the patient's cousins developed cardiovascular symptoms in their mid‐twenties. No additional details about their condition were available.

The patient was first admitted to a hospital at the age of 15 following an episode of syncope accompanied by a brief loss of consciousness. He also reported occasional left‐sided stabbing chest pain.

During this initial hospitalization, the ECG was unremarkable for any conduction or repolarization abnormalities. There were no signs of left ventricular hypertrophy according to patient records (Sokolow–Lyon index [9]). Clinical examination was unremarkable. The patient was hemodynamically stable and euvolemic. Cardiac auscultation revealed no murmurs, rubs, or gallops. Echocardiographic examination identified HCM with a normal LVEF and reduced ejection volume. The septal thickness was 14 mm. Holter monitoring revealed tachycardic episodes with up to 148 bpm, no premature ventricular contractions (PVCs), three supraventricular contractions (SVCs), and discrete ST segment elevations in lead I and lead II, with no ST segment depression. According to patient records, high‐sensitivity Troponin T (hsTnT) and NT pro‐brain natriuretic peptide (NTproBNP) were within the normal range. Bicycle stress test revealed reduced exercise capacity, characterized by a pathological increase in heart rate, reaching 45% of the expected level for the patient's age group. The test was terminated prematurely due to the patient's inability to continue exercising. Immediately following the test, a loud systolic murmur was auscultated at the aortic valve position.

Treatment with metoprolol succinate 23.75 mg once daily was initiated to manage tachycardic episodes, followed by regular follow‐up appointments. Additionally, the patient and his parents were advised to limit physical activity to a moderate level. Over the following years, the dose of metoprolol succinate was gradually increased to 95 mg in the morning and 47.5 mg in the evening.

**FIGURE 1:**
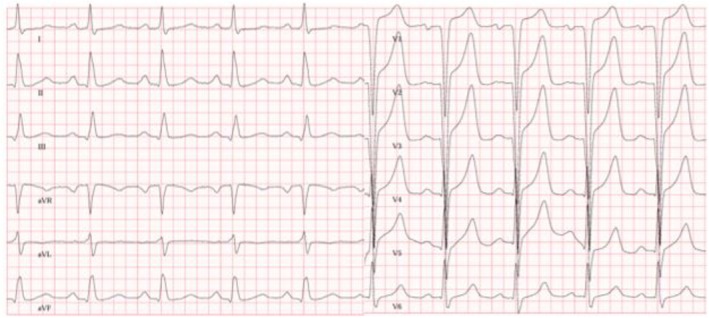
Initial ECG of the patient with positive Sokolow–Lyon index indicating cardiac hypertrophy.

The patient first presented to our hospital at the age of 27 for EMB due to the progression of left ventricular wall thickness, with symptoms according to NYHA II as follows: occasional left‐sided chest pain, dizziness, and reduced physical endurance. The ECG revealed a positive Sokolow–Lyon index and frequent tachycardic episodes, with a heart rate of up to 95 beats/min at rest (Figure 1 [9]). At the initial examination, the patient was hemodynamically stable and euvolemic, with cardiac auscultation revealing no murmurs, rubs, or gallops. HsTnT was elevated to 56 ng/dL and NTproBNP to 2008 ng/dL.

**FIGURE 2:**
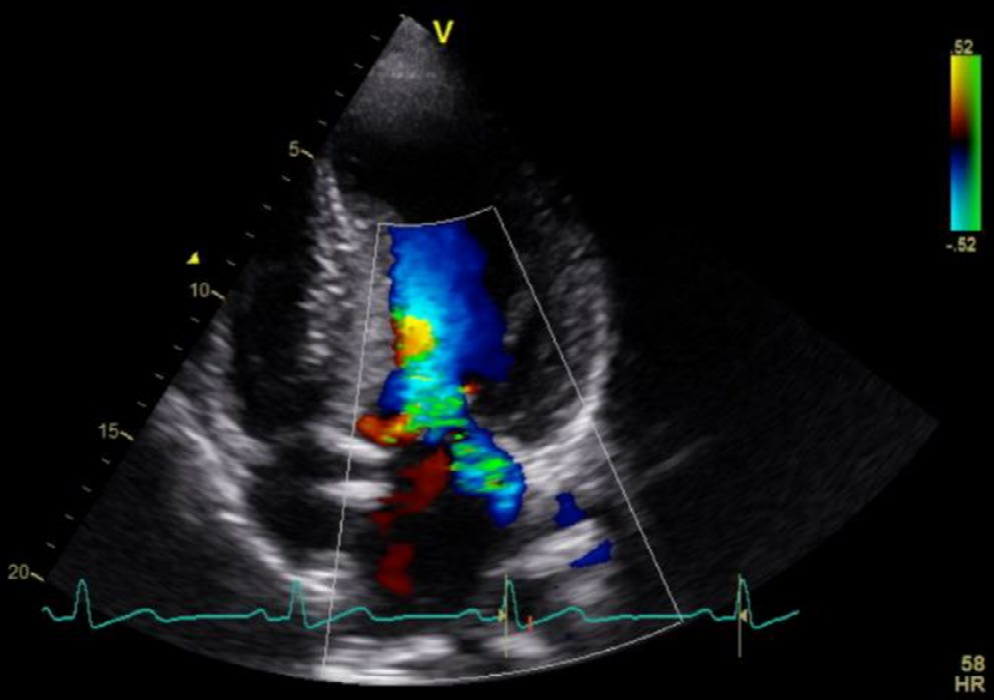
Transthoracic color duplex echocardiography of the heart in the apical three‐chamber view showing a regurgitant jet into the left atrium, consistent with mitral insufficiency, Grade 3.

Echocardiographic examination revealed a normal LVEF of 70%, with a significantly increased interventricular septum thickness in diastole (IVSd) of 25 mm and posterior wall thickness in diastole (PWd) of 18 mm. The LVOT gradient was 130 mmHg. SAM of the mitral valve was prominent. The mitral insufficiency, initially classified as Grade 1, progressed to a severe Grade 3 (Figure 2).

Due to claustrophobia, the patient declined CMR imaging. To assess a potential storage disease, an EMB was performed. In addition to the EMB, the patient underwent coronary angiography in preparation for a potential TASH. He was also referred to the electrophysiology department to be evaluated for an implantable cardioverter defibrillator (ICD), given his ESC HCM Risk‐SCD score of 7.56%. The EMB tested negative for cardiotropic viruses. Storage diseases, such as amyloidosis and glycogenosis, as well as acute or chronic myocarditis, were ruled out. Histological analysis revealed interstitial fibrosis and moderate signs of cardiomyocyte hypertrophy. Additionally, a myocyte disarray and focal myofibrillar loss could be seen. Genetic testing revealed a variant of unknown significance in the gene MYBPC3. In summary, the EMB findings were consistent with a diagnosis of primary HCM.

Based on these findings, the patient was presented to the heart team. Given the imminent approval of mavacamten in Europe, a mutual decision was made to pursue a conservative approach and await the availability of mavacamten for a therapeutic trial instead of pursuing myectomy and mitral valve surgery or transcoronary ablation of septal hypertrophy (TASH). Prior to initiating mavacamten, CYP2C19 testing was conducted, confirming that no dose adjustment was required for this patient.

**FIGURE 3:**
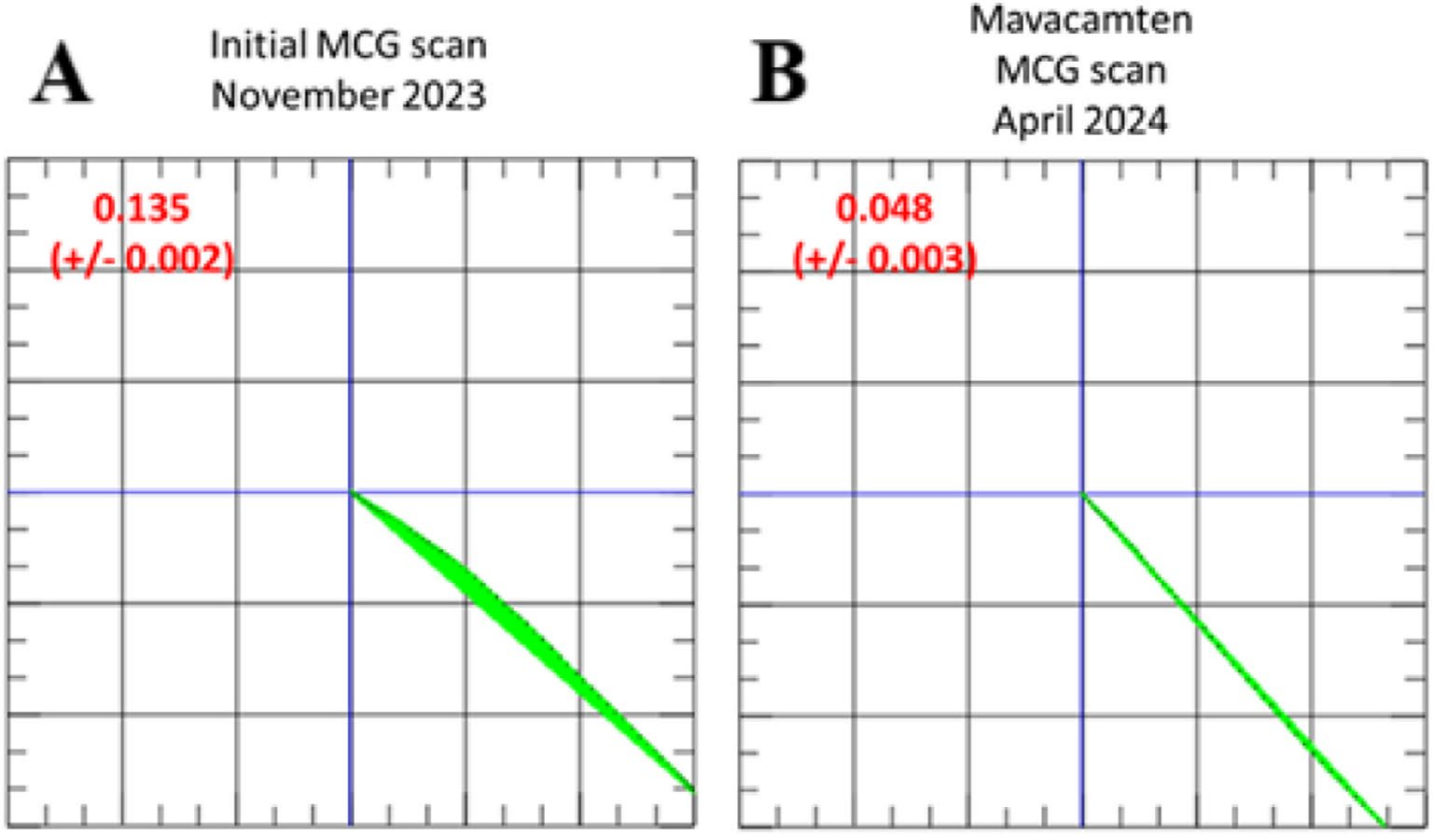
(A) Initial MCG vector of 0.135 (± 0.002). According to our previous findings, the MCG vector, which is directed toward the lower right quadrant, is considered pathological (≥ 0.051 [5]). (B) MCG vector after 5 months of mavcamten treatment of 0.048 (± 0.003). The MCG vector decreases, moving toward a score that is considered normal (< 0.051) based on our previous findings [5] and suggestive of a response to therapy.

In addition, we performed MCG to determine the electromagnetic vector of the heart at baseline and during treatment. We recently demonstrated that MCG can be used to detect cardiomyopathy and treatment response in inflammatory cardiomyopathy and amyloidosis [5, 10]. A threshold of ≥ 0.051 for the identification of cardiomyopathy was established in our previous work [5], classifying this patient's MCG vector of 0.135 in the lower right quadrant as distinctly pathological (Figure 3A).

## 3 Method: Magnetocardiography

MCG uses quantum technology to measure the electromagnetic field of the heart [5].

This electromagnetic field is a result of the generation of action potentials in cardiomyocytes through ion movement across cell membranes. The intensity and direction of this magnetic field are determined by the ionic flux within the cells. Typically, the strength of the cardiac electromagnetic field ranges from 10^−15^ to 10^−11^ Tesla [5].

**FIGURE 4:**
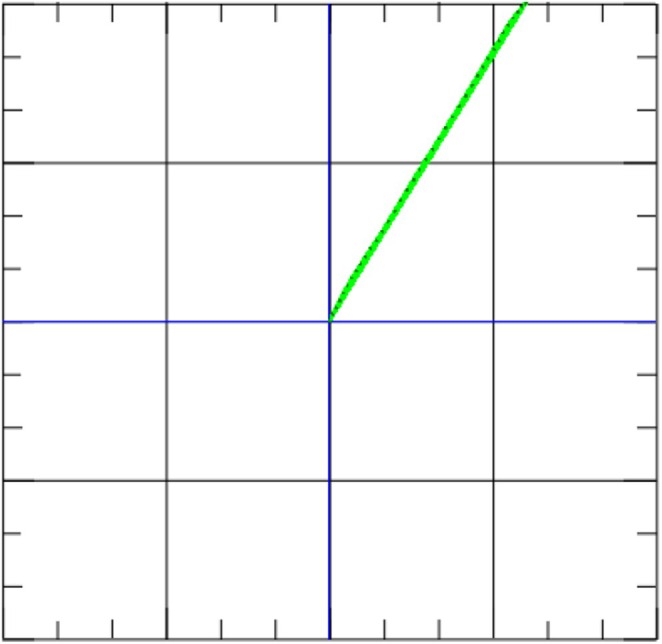
MCG vector of a healthy subject. The narrow MCG vector (green plane) is located in the right upper quadrant with an MCG vector of < 0.051, which is considered physiological [3].

The MCG system employs an array of 64 superconducting quantum interference devices (SQUIDs), which are highly sensitive magnetic sensors. To minimize interference from external electromagnetic signals, the measurements are conducted within a shielded room. SQUIDs are capable of detecting changes in the heart's magnetic field throughout the cardiac cycle, correlating these variations with the QRS complex. Multiple frequency filters are applied to eliminate electromagnetic noise. This method allows a three‐dimensional assessment of the magnetic field, making it possible to calculate a composite vector that represents the heart's primary electrical axis. In assessing inflammatory cardiomyopathies, particular focus is given to the T‐wave action potential vector and its peak, described by the T‐beg to Tmax interval. Our previous studies have shown that an MCG vector (T‐beg–Tmax) ≥ 0.051 is indicative of pathological conditions [5]. Also, we demonstrated that the direction of a normal vector is located in the right upper quadrant (Figure 4 [5]). The MCG vector is not exclusive to inflammatory cardiomyopathy, but may also be altered in other cardiomyopathies, such as ischemic cardiomyopathy or HCM. Therefore, it is a sensitive screening tool for the detection of cardiomyopathies overall.

## 4 Outcome and Conclusion

At both the 2‐week and 2‐month follow‐up appointments, there were no significant changes in the echocardiographic parameters, with the hyperdynamic LVEF remaining at about 75%. HsTnT improved from 56 to 11 ng/dL and NTproBNP from 2008 to 765 ng/dL. The patient reported no side effects of mavacamten, such as dizziness, fainting, chest pain, or reduced urine output.

Five months after the initiation of mavacamten, follow‐up MCG revealed a vector remaining in the right lower quadrant, which is considered still abnormal [5], but with a decreasing value from 0.135 to 0.048 (Figure 3B). This finding is biologically plausible, as structural changes in HCM are not entirely reversed during mavacamten therapy, according to current evidence in the literature. Improvement was further confirmed by echocardiographic assessment, showing a decrease in the LVOT gradient from > 130 to 39 mmHg, while IVSd remained at 25 mm and PWd at 18 mm. Consequently, the severity of mitral insufficiency improved from Grade 3 to Grade 1. The patient improved clinically from NYHA Class II to NYHA Class I. HsTnT decreased further from 11 to 6 ng/dL, while NTproBNP stayed at 768 ng/dL.

Given the observed clinical improvement, there was no more indication for SRT and mitral valve surgery. Follow‐up appointments were scheduled every 3 months in the cardiology outpatient clinic. At the most recent follow‐up, 8 months post treatment initiation, the patient did not report any additional changes in symptoms or side effects from his medication.

This case demonstrates the potential of mavacamten as a noninvasive therapeutic option that significantly improves patient outcomes by reducing LVOT obstruction. Compared to traditional interventions such as myectomy or TASH, which carry procedural risks, mavacamten offers a safe alternative.

Given the heterogeneous nature of HCM and the variability in treatment response, there is a growing need for rapid and reliable diagnostic tools to evaluate therapeutic effectiveness. MCG was able to detect early therapy response in this patient and emerges as a promising solution, offering quick and objective data with a scanning time of 60 s and a reading time of approximately 5 min. This enables real‐time assessment of cardiac electromagnetic activity, allowing clinicians to detect early treatment response to optimize care and potentially improve patient outcomes more efficiently.

## 5 Discussion

This case highlights the promising role of mavacamten in HCM, offering a targeted, noninvasive therapeutic option through decreasing LVOT obstruction [11, 12, 13]. Given the dynamic nature of HCM and the variability in patient response to treatment, there is a need for objective evaluation of therapeutic efficiency.

Echocardiography and CMR are considered the gold standards for assessing HCM [2]. MCG is very unlikely to replace either of the two primary diagnostic methods. However, it serves as a valuable complementary method by providing an objective score that minimizes inter‐reader variability. This reduces the learning curve and the level of experience required for accurate interpretation. Additionally, MCG data acquisition takes only 1 min, with interpretation typically completed in about 5 min, making it an efficient tool for rapid screening [5]. Being side effect free, it can be applied with a low threshold and serially in short intervals, even in vulnerable populations including children, pregnant women, or patients with kidney disease. MCG enables physicians to assess myocardial electromagnetic activity in real time, allowing for early treatment adjustments that may enhance therapy responsiveness. While this technology involves higher technical costs, these could be offset by reduced personnel expenses, an increased volume of patient data acquisition per day, and, most importantly, the potential for improved clinical outcomes in the future.

In comparison with prior literature, it is evident that this case led to similarly favorable outcomes, particularly an improvement in quality of life, accompanied by a lower NYHA classification, reduction of LVOT gradient, while maintaining a stable LVEF [14, 15, 16, 17]. Few cases have been reported where the therapy had to be discontinued due to side effects, such as worsening hypertension and an LVEF below 50% [14, 17, 18]. Ultimately, the safety of mavacamten may be tested further in other conditions, including other comorbidities, ethnic diversity, or long‐term use [15, 16, 17].

In summary of this case, continuous research and clinical trials are needed to: (a) validate the effectiveness of MCG as a diagnostic tool for screening HCM and monitoring treatment response and (b) investigate whether improvements in the electromagnetic vector in HCM patients treated with mavacamten are associated with a reduced risk of arrhythmias—a leading cause of SCD in this cardiomyopathy.

## Author Contributions

**Phillip Suwalski:** methodology, supervision, visualization, writing – original draft, writing – review and editing. **Gamze Satilmis:** writing – original draft, writing – review and editing. **Markus Reinthaler:** writing – review and editing. **Ulf Landmesser:** writing – review and editing. **Bettina Heidecker:** supervision, validation, writing – review and editing.

## Ethics Statement

The ethics committee of the Deutsches Herzzentrum der Charité (DHZC) Universitätsmedizin Berlin approved the study (EA4/193/17).

## Consent

Written informed consent was obtained from the patient for the publication of this case report.

## Conflicts of Interest

PD Dr. med. Heidecker is an inventor on patents that use RNA for diagnosis of myocarditis. Patent protection is in process for MCG for diagnosis and measurement of therapy response in inflammatory cardiomyopathy. The remaining authors have no disclosures to report.

## Data Availability

Data from the study can be made available upon reasonable request from the principal investigator Prof. Dr. med. Bettina Heidecker in a de‐identified form. A data use agreement must be signed before access to data. Access is only granted to academic staff.
